# Complete genome of *Lactococcus lactis* isolate S11-599 from the brain of a silver carp (*Hypophthalmichthys molitrix*) during a mass mortality event in the Mississippi river

**DOI:** 10.1128/mra.00503-25

**Published:** 2025-09-08

**Authors:** Noor-Ul Huda, Caitlin E. Older, Cynthia Ware, Taylor I. Heckman, Divya Rose, David P. Marancik, Lester H. Khoo, Matt J. Griffin

**Affiliations:** 1 Department of Pathobiology and Population Medicine, College of Veterinary Medicine, Mississippi State University 70204, Mississippi State, Mississippi, USA; 2 Thad Cochran National Warmwater Aquaculture Center, Delta Research and Extension Center, Mississippi State University 7705, Stoneville, Mississippi, USA; 3 Department of Pathobiology, School of Veterinary Medicine, St George's University 14339, True Blue, Grenada, West Indies; 4 Warmwater Aquaculture Research Unit, Agricultural Research Service, United States Department of Agriculture, Stoneville, Mississippi, USA; 5 Department of Wildlife, Fisheries and Aquaculture, College of Forest Resources, Mississippi State University 237138, Mississippi State, Mississippi, USA; Montana State University, Bozeman, Montana, USA

**Keywords:** *Lactococcus lactis*, silver carp, *Hypophthalmichthys molitrix*, meningoencephalitis

## Abstract

The complete genome of *Lactococcus lactis* isolate S11-599 is presented, recovered from the brain of a silver carp (*Hypophthalmichthys molitrix*) during a fish mortality event affecting invasive carp in the Mississippi River.

## ANNOUNCEMENT

*Lactococcus lactis* is a gram-positive, generally commensal bacterium with probiotic potential (1, 2). However, it has been implicated in infections in humans (3, 4), cows (5), birds (6), and aquatic animals (7). In September 2011, fish mortality was reported along the Mississippi River, predominantly affecting invasive carp. Isolate S11-599 was recovered from the brain culture of a silver carp (*Hypophthalmichthys molitrix*) collected at Tunica Cutoff, MS, plated on Mueller–Hinton agar with 5% sheep’s blood at 26°C, and initially identified as *L. lactis* subsp. *lactis* by biochemical and molecular analyses (8).

Cell pellets of S11-599 were resuspended in Zymo DNA/RNA Shield and shipped to Plasmidsaurus Inc. for genomic DNA extraction using the Zymo Quick-DNA Miniprep Plus Kit, and sequencing was performed without additional size selection/shearing. Long-read sequencing (Oxford Nanopore Technologies [ONT] plc, Oxford, UK) was performed using Rapid Barcoding Kit V14 (SQK-RBK114.96) on PromethION with R10.4.1 flowcell and simultaneous quality filtering (q ≥10) with the super-accurate basecalling mode (v4.2.0). Default parameters were used for all software unless otherwise noted. Data (N_50_: 7,506 bp, 1.4 Gb) were subset to 100× coverage with Filtlong (v0.2.1, --length_weight 10, https://github.com/rrwick/Filtlong) before assembly with Canu (v2.2, genome size = 2.5 m) (9).

Illumina libraries were prepared using the ExpressPlex Kit (SeqWell), sequenced on NextSeq 2000 (2 × 150 bp, 492.8 Mb). Data were quality filtered with a 4-bp sliding window cutoff of Q20, a minimum sequence length of 75bp, and adapter removal in trimmomatic (v0.39) (10), and polish the ONT assembly via polypolish (v0.6.0) (11), using bwa (v0.7.17) (12) to generate the intermediate BAM files. Vim (v8.2) was used to identify and manually trim sequence overlap by reorienting the contig to the *gyrB* gene and then mapping the long reads to the contig using minimap2 (v2.2) (13) to verify contiguous coverage across the trimmed region.

The genome (100× ONT and 156× Illumina) was annotated through NCBI’s PGAP (v6.8) (14). Whole-genome average nucleotide identity (ANI) was computed by FastANI (v0.1.3) in Kbase (https://www.kbase.us/) (15). Digital DNA–DNA hybridization estimations (dDDH) were made by GGDC 4.0 with formula 2 through the Type (Strain) Genome Server (https://tygs.dsmz.de/) (16). Plasmids were characterized using Plascad (v1.17) (17), and genome completeness was assessed using CheckM (v1.2.3) (18).

Sequencing statistics and genomic features of *L. lactis* S11-599 are provided in Table 1. The genome consists of one circular 2,590,798 bp chromosome (NCBI accession CP170449) and a mobile (MobB), nonconjugative 15,601 bp plasmid (CP170450). A 6,186 bp sequence containing genes involved in metabolic or structural processes was present in five tandem copies and appears to be unique to this isolate (Table 1). Long reads were mapped to this repetitive region using minimap2 (v2.2) to verify reads spanning the five repeats and flanking sequence. The isolate showed 97.9% ANI and 82.2% dDDH similarity to the type strain of *L. lactis* subsp. *lactis* (ATCC 19435; GCF_039544895.1/), and 97.4% ANI and 79% dDDH similarity to the type strain of *L. lactis* subsp. *hordniae* (NBRC 100931; GCF_039544895.1). These values suggest that isolate S11-599 recovered from a fish mortality event involving a destructive invasive species may represent a novel *L. lactis* subspecies.

**TABLE 1:** Genome features and functional annotation of genes within a five-copy, 6,186-bp tandem repeat region in the genome of *L. lactis* isolate S11-599

Feature	Sequencing and genome metrics
No. of raw reads (ONT)	373,121
No. of filtered reads	12,045
% Retained	3.23
No. of raw reads (Illumina)	1,549,821
No. of filtered reads	1,433,154
% Retained	92.46
Genome size (bp)	2,606,399
Completeness (%)	99.5
No. of contigs	2
GC content (%)	35
No. of genes	2,579
No. of CDSs	2,488
No. of rRNA genes (5S, 16S, and 23S)	7, 6, and 6
No. of tRNAs	68
No. of ncRNAs	4

Gene/feature	Length (bp)	Putative function
*dapD*	771	Key enzyme of the DAP pathway; involved in lysine and peptidoglycan biosynthesis
*ecfA1*	834	ATPase subunit of energy-coupling factor (ECF) transporter complex; hydrolyzes ATP to energize micronutrient uptake (e.g., vitamins and metals)
*ecfA2*	867	ATPase subunit of ECF transporter; works with *ecfA1* to drive nutrient uptake via ATP hydrolysis
*ecfT*	801	Transmembrane subunit of ECF transporter; forms the channel and couples ATP hydrolysis to nutrient transport
*N*-acetyltransferase domain-containing protein	489	Transfers acetyl groups to proteins or substrates, aiding in modification or metabolic regulation
*N*-acetyldiaminopimelate deacetylase	345	Part of the DAP pathway; involved in lysine and peptidoglycan synthesis
Thiol-disulfide isomerase	612	Facilitates proper protein folding through the formation, breakage, or rearrangement of disulfide bonds
Tyrosine-type recombinase/integrase	738	Catalyzes site-specific DNA recombination, enables genomic rearrangements, and DNA integration

## Data Availability

The complete genome sequence of *L. lactis*, isolate S11-599, has been deposited in NCBI under BioProject accession no. PRJNA1165287. The raw reads used to generate this assembly can be found at SRR33387906 and SRR34591821.
